# White matter differences in motor and affective-motivational networks of pain-indifferent carriers of the R221W mutation

**DOI:** 10.1016/j.ynpai.2026.100211

**Published:** 2026-03-28

**Authors:** Arnas Tamasauskas, Irene Perini, Jan Minde, Simon S. Keller, Nicholas Fallon, Bernhard Frank, India Morrison, Andrew Marshall

**Affiliations:** a University of Liverpool, Foundation Building, 765 Brownlow Hill, Liverpool L69 7ZX, United Kingdom; b Linköping University, SE-581 83 Linköping, Sweden

**Keywords:** Congenital pain insensitivity, Neuropathy, Genetic conditon, Fixel analysis, Graphtheory, Network based statistics, Diffusion tensor imaging, White matter integrity, Region of interest analysis, Nociceptive network, Saliance network

## Abstract

The R221W mutation on the nerve growth factor gene results in reduced peripheral C-nociceptor density and behavioural indifference to painful stimuli. While functional neuroimaging has revealed altered cortical and sub-cortical pain processing in R221W carriers, structural white matter changes remain unexplored and may suggest an anatomical basis of symptoms. Heterozygous R221W carriers’ (n = 11) and matched controls’ (n = 11) diffusion MRI data were compared using fixel-based analysis, and complimentary edge and node analyses using graph theory and network-based statistics. Whole-brain and region of interest (ROI) fixel-based analyses revealed significantly reduced fibre density and fibre-bundle cross-section in brainstem motor tracts of R221W carriers, encompassing the corticospinal pathways, corona radiata, external capsule, cerebellar peduncles, and pontine crossing (p < 0.05). Graph theory analysis of pain-processing ROIs demonstrated significantly reduced node degree and betweenness centrality in the Left Anterior Cingulate Cortex (ACC) of R221W carriers, indicating structural isolation of this affective-motivational hub. Network-based statistics identified significantly stronger connectivity between the Right Thalamus and Right ACC in R221W carriers, in contrast to weaker connectivity connecting the Right Thalamus to the Left ACC and Left Insula (p < 0.05). These findings indicate potentially reduced brainstem motor tract integrity and altered cortical network topology, specifically the structural isolation of the Left ACC. This, alongside potentially compensatory right-sided thalamo-cortical connectivity and preserved sensory afferent pathways, supports a model of R221W pain indifference as motor under-reactivity rather than sensory insensitivity.

## Highlights

• R221W carriers’ central pain pathways are potentially impacted by chronic underactivity.
• Motor pathway deficits, not sensory loss, underpin R221W pain indifference.
• R221W carriers show reduced fibre density in key brainstem motor tracts.
• Inter-hemispheric connectivity appears to be disrupted in R221W carriers.
• Right-sided thalamo-cortical connectivity appears to be stronger in R221W carriers.

## 1 Introduction

For most of us, pain is an inescapable part of being. However, for rare individuals with reduced pain sensitivity, pain is just a subtle, insignificant sensation. Acquired peripheral neuropathies usually result in slightly reduced pain sensitivity (Bierhaus et al., 2004, Koike et al., 2010), such as reduced reactivity to pinpricks. Congenital Insensitivity to Pain (CIP) (Lischka et al., 2022, Schon et al., 2020) syndromes are more severe, resulting in individuals sustaining significant bodily harm and letting their wounds go untreated, particularly broken bones and fractures (Lischka et al., 2022). The prevalence of CIP is unknown; some studies have reported the prevalence of 1 in 25,000(Schon et al., 2020) but the origin of these figures in scientific literature is unclear.

CIP has been seen in people with mutations in SCN9a (Cox et al., 2006), CLTCL1 (Nahorski and Al-Gazali, 2015), NTRKI (Indo, 2001), and R221W (Indo, 2012) genes. The R221W gene mutation in particular results in reduced nerve growth factor neuropeptide secretion. This reduction results in a lower density of unmyelinated C-nociceptor afferent nerves in the skin – a type of peripheral neuropathy (Larsson et al., 2009). C afferents carry somatosensory information of mechanical, thermal, and chemical nociception from the body’s periphery to the central nervous system (Ackerley and Watkins, 2018). Recent research into R221W carriers has shown that this reduction in unmyelinated C-afferents results in indifference to pain (Perini et al., 2016, Nagasako et al., 2003). These afferents form part of nociceptive pathways and the reduced activity translates to under-activation of pain-related brain areas, possibly linked to carriers’ observed behavioural under-reaction to pain. While R221W heterozygote carriers are able to report subjective thermal pain thresholds to the same degree as non-carrier controls, they lower and slower reported motivational urge to escape painful heat stimulation compared to controls (Perini et al., 2016). These behavioural responses correlated to an under-activation of the middle part of Anterior Cingulate Cortex (ACC) and anterior insula during a functional Magnetic Resonance Imaging (MRI) motor response task involving heat pain stimulation (Perini et al., 2020). These findings imply that altered peripheral nerve density is related to altered pain processing in the heterozygote R221W carrier population. As research has shown that ACC and anterior insula areas are usually linked to a participant’s motivation to respond; it was hypothesized that R221W carriers showed increased reliance on the sensorimotor cortex instead (Perini et al., 2020).

Functional MRI activation changes were also seen in people with acquired peripheral neuropathy. In diabetic neuropathy, patients can present with hyperalgesia or hypoalgesia sensory signs (Zang et al., 2023, Dyck et al., 2000). When these groups were compared in a heat pain functional MRI task, people with hypoalgesia exhibited reduced functional connectivity between the insula, amygdala and ACC (Wilkinson et al., 2020). This reduced functional connectivity correlates with white matter connectivity changes observed in people with diabetic neuropathy. Using structural MRI, people with diabetic neuropathy were shown to have reduced white matter pathways in the sub-cortical pain processing areas including the thalamus, insula, and ACC compared to healthy controls (Zhang et al., 2020, Xiong et al., 2022). In cases of peripheral neuropathy, it is difficult to discern whether these structural and functional changes in the brain are due to under-activated peripheral-central nerves, or whether these changes are due to neurological damage from the conditions that have caused neuropathy (Timtim et al., 2022). For example, acquired neuropathy due to diabetes is commonly associated with small vessel disease or chronic inflammation (Timtim et al., 2022, Dudek et al., 2020, Sanahuja et al., 2016, Shukla et al., 2017). Cases of CIP allow for investigation of central structural differences in peripheral neuropathy without metabolic or inflammatory confounders.

To understand central nervous system alterations in R221W carriers, diffusion tensor imaging (DTI) can provide insight into structural connectivity anomalies (Assaf and Pasternak, 2008). However, DTI modelling has historically been limited by statistical, practical and theoretical uncertainty in how to approach crossing fibres (Farquharson et al., 2013). Fixel-based analysis provides a novel technique that quantifies fibre orientation distribution in individual voxels into “fixels” (Dhollander et al., 2021, Raffelt et al., 2015) (a fibre equivalent of voxels), which represent individual fibre populations within a given voxel, allowing for increased specificity over voxel-wise measures. Using fixel-based analysis, precise fibre bundles within crossing-fibre voxels can be compared using group-wise analyses of three metrics: fibre density (FD), fibre-bundle cross-section, and a combined measure of fibre density and fibre-bundle cross-section (FDC) (Raffelt et al., 2012), which may reflect different aspects of neural damage. Fixel-based analyses have been shown to be more sensitive to pathological alterations of white matter in various neurological disorders compared to analysis of conventional DTI scalar metrics (Li et al., 2020, Ahmadi et al., 2024, Oh et al., 2021). Whole-brain fixel analysis was performed as an unbiased exploratory assessment, while region of interest (ROI) analyses were anatomically constrained and hypothesis-driven to increase sensitivity within predefined networks implicated in nociceptive and salience processing. Building on functional MRI research of R221W carriers (Perini et al., 2020), the ROI for this study was defined to include cortical and subcortical salience processing areas the wider nociceptive areas that they project to: left and right sensory cortices, left and right ACC, left and right insula, left and right thalamus, and brainstem (Sun et al., 2023, Lançon and Séguéla, 2023, Brooks and Tracey, 2007, Jensen et al., 2016, Groh et al., 2018, Stroman et al., 2018). As this was a first investigation of white matter tracts in a CIP cohort, the exploratory whole-brain analysis provided supplementary exploration of areas that are not directly related to pain processing.

Alongside fixel-analysis, ROI areas were analysed using graph theory – a computational neuroscience analysis that provides information of how well vertices of converging connections, referred to as ‘nodes’, are integrated, segregated, or centralised in brain networks (Sporns, 2012, Lenoir et al., 2021, Wada et al., 2017, Liu et al., 2013). In graph theory, nodes are vertices of converging connections linked by edges which represent connectivity of nodes in a network, allowing analysis of how a brain network is organised. Graph theory provides a complementary approach to fixel-analysis, as it can be used to investigate how focal changes, that are identified by fixel-analyses, relate to topological differences in pain processing networks. These topological differences reflect the magnitude and importance of nodes and edges connecting them. Edges can additionally be analysed on a group level using Network Based Statistics (NBS) (Zalesky et al., 2010). This method utilises a nonparametric permutation approach combined with cluster-based thresholding to identify suprathreshold links. NBS has been instrumental in identifying edge connectivity differences, that were not previously found in graph theory research, in peripheral neuropathy groups. Graph theory has identified a potential link between acquired painful diabetic neuropathy and increased global and local efficiency in the ACC and reduced in the insula (Teh et al., 2021, Axelsen et al., 2025, Yang et al., 2020). Research using NBS on a painful diabetic neuropathy group found an almost opposite effect – the insula had reduced connectivity to cortical pain processing areas (Chao et al., 2022, Chao et al., 2023). The contrasting NBS and graph theory findings on diabetic neuropathy participants’ insula connectivity illustrates how both methods identify complementary but distinct topological changes that provide nuanced connectivity findings. While these investigations provide information about the structural connectivity differences in chronic pain groups (Chao et al., 2022, Chao et al., 2023), there is no investigation of the opposite sensation – neuropathy with hypoalgesia. Graph theory and NBS together can provide a robust investigation into R221W carriers’ global brain network reorganisation, and provide wider white matter connectome context to fixel-based analysis.

The aim of the present study was to identify how structural brain networks correlate to congenitally reduced C-nociceptive fibres. Fixel-based whole-brain and ROI analyses were carried out to investigate CIP individuals’ brain structural white matter integrity and provide structural context for previous functional MRI findings. Graph theory and NBS analyses can shed light on how focal changes correlate to network organisation in shaping somatosensory signalling and perceptual experience. It can also pave the way for new approaches for interrogating this role, and of further testing and interpreting effects of CIP on central nervous system phenotypes. As there is no previous DTI research into congenital pain insensitivity, the hypotheses of this study were based on functional MRI R221W research (Nagasako et al., 2003) and DTI research of another peripheral neuropathy group – diabetic neuropathy (Zhang et al., 2020). We hypothesised that R221W carriers would show reduced fibre density and cross section in the corticospinal and spinothalamic tract when compared to healthy controls. We hypothesised that ROI analysis would show reduced integrity of R221W carriers’ supplementary pain processing white matter tracts – cingulum, and anterior limb of internal capsule. We additionally hypothesised that in R221W carriers, graph theory and NBS would show integration, segregation, and centrality to be increased in ACC and Insula but reduced in sensory cortices.

## 2 Methods

### 2.1 Participants

The study protocol was in accordance with Declaration of Helsinki and approved by Gothenburg University Hospital ethics board as a collaboration with Umeå University. A group of eleven heterozygous R221W carriers and eleven control participants were recruited for this study. R221W carriers were recruited based on pedigree (at least one carrier parent), and the presence of the mutation ((an arginine-to-tryptophan substitution on one allele of the beta subunit of the NGF gene) later confirmed by DNA sequencing. Arthropathies in R221W carriers show a progressive trend, with mild or unaffected carriers showing clinical symptoms decades later. Both groups were recruited in Sweden and the carriers lived in a geographically dispersed area of northern Sweden.

The R221W carriers group and control group were age, sex, and education matched. The mean age of R221W carriers was 36.2 years, and the mean age of matched controls was 35.8 years. Both groups had 7 female and 4 male participants. Both carriers and controls had 12 years of formal education. Some of the heterozygous carriers exhibited subclinical signs of arthropathies, carpal tunnel, and painless fractures. Cognitive functions and basic reflexes were normal in carriers, and they did not differ from controls on the ability to discriminate noxious and innocuous thermal stimulation as assessed by threshold testing (Perini et al., 2020).

### 2.2 MRI acquisition

Data was acquired using 3 T General Electric scanner with 32-channel head coil at the Umeå Centre for Functional Brain Imaging, Umeå University Hospital (Umeå, Sweden). Diffusion acquisition consisted of 32 directions at *b* = 1,000 s/mm–2 and six *b* = 0 s/mm^−2^ volumes acquired at matrix size = 256 × 256, slices = 64, field of view = 250 × 250 mm. The Repetition Time (TR) was 8 s, and Echo Time (TE) was 81.4 ms, with a total acquisition time of 5 min and 4 s. This resulted in an acquired voxel size of 0.98 × 0.98 × 2.0 mm. T1-weighted structural images were acquired with the following parameters: matrix size = 505 × 412, voxel size 0.4883 x 0.4883 x 1 mm, field of view = 246 × 201 mm. This resulted in a voxel size of 0.49 × 0.49 × 1.0 mm. The sequence used a TR of 8.2 ms, TE of 1.5 ms, and a flip angle of 12°. The total acquisition time was approximately 6 min.

### 2.3 Pre-processing

As DTI was acquired using single phase-encoding (anterior-posterior), a generative Synb0-DISCO (Schilling, 2019) algorithm was used to synthesise a reverse (posterior-anterior) phase-encoded image. The acquired DTI and synthesised DTI images were then used for preprocessing using FMRIB software library (FSL) (Smith and Jenkinson, 2004, Jenkinson and Beckmann, 2012). FSL was first used with the TOPUP command to estimate and correct susceptibility induced distortions, and then EDDY command to correct for eddy currents and head movement. MRtrix (Tournier et al., 2012) toolbox (*dwibiascorrect*) was used for bias field correction and global intensity normalisation across subjects. Motion parameters were examined to ensure there was no significant drift or head motion. The mean frame-wise displacement was approximately 0.11 mm, indicating minimal head movement during the scan. The maximum absolute displacement was 0.56 mm.

The T1-weighted image of each participant was pre-processed using MRTrix (Tournier et al., 2012) to create a T1-weighted image with five different tissue types – cortical grey matter, subcortical grey matter, white matter, cerebrospinal fluid, and pathological tissue. For each participant, the segmented T1-weighted image was registered to their pre-processed DTI image.

### 2.4 Computation of Fixel metrics

Recommended methodology was followed for Fixel metrics generation (Raffelt, 2017). Pre-processed DTI were bias field corrected using Advanced Normalization Tools (Avants et al., 2009). The following steps were then executed using MRtrix (Tournier et al., 2012) toolbox: DTI images were upsampled and Fiber Orientation Distribution (FOD) estimations was performed with a multi-tissue constrained spherical deconvolution algorithm (*SS3T-*CSD) (Jeurissen et al., 2014) using the group average white matter response function. These FODs for each tissue were bias field corrected and had global intensity normalisation (*mtnormalise*) applied across subjects. An FOD template was created from all subjects, and wraps were computed to register each participant to the templates. A Fixel mask was then created from the FOD template. Each individual’s segmented T1-weighted images were also warp registered to the FOD template, and were then used to create an anatomically constrained mask.

For each subject, FOD images were segmented to identify the number and orientation of Fixels in each voxel, as well as estimating apparent FD in each Fixel. Once the directions of all subject image Fixels were reoriented based on a Jacobian matrix, the FD value of each subject’s Fixels were assigned to the corresponding Fixel in the FOD population template. Fibre cross-section was computed from warps generated in previous steps, which was then used to compute a combined FDC metric. Log metric was calculated for fibre cross-section scores to ensure normal distribution (LogFC).

### 2.5 Whole-brain Fixel-based analysis

Whole-brain fibre tractography was performed using a probabilistic algorithm − Second-order Integration over Fiber Orientation Distributions (iFOD2) (Tournier et al., 2010) with an anatomically constrained mask. Ten million streamlines were generated using the recommended tracking angle (22.5°) and lengths (min 10; max 250). This was followed by Spherical-deconvolution Informed Filtering of Tracks (SIFT) (Smith et al., 2013) to control for track thickness overestimation and straight track density bias. For reduction of streamlines using SIFT, a factor of ten was selected as it is a heuristic guideline outlined in MRTrix (Tournier et al., 2019) and commonly used in Fixel research (Rudolph et al., 2024). A Fixel-Fixel connectivity matrix was then generated from one million streamlines. Fixel connectivity filtering using the connectivity matrix was applied to FD, LogFC, and FDC to reduce noise (Raffelt et al., 2015). Permutation testing was then performed on these metrics with family-wise error rate correction using the threshold-free cluster enhancement (Jenkinson et al., 2002). Resulting significant Fixel plots were converted to voxels and used as masks to reduce the whole-brain tractogram to ten thousand streamlines using SIFT for visualisation of white matter streamlines going through significant Fixel areas. These significant Fixel plot streamlines were then registered to a John Hopkins University (JHU) ICBM-DTI-81 white-matter labels atlas (Mori et al., 2005) using FSL Linear Image Registration Tool (Andersson et al., 2007) and Non-Linear Registration Tool (Jenkinson and Beckmann, 2012) to identify associated anatomical pathways.

### 2.6 ROI Fixel analysis

ROI analysis was undertaken alongside whole brain analysis to increase sensitivity for findings in a small sample, and to create Fixel parcellated ROI connectomes to be used in graph theory analysis. ROI selection was based on prior task-based functional MRI (fMRI) and by literature consistently implicating distributed cortical and subcortical regions in nociceptive and salience processing (Jensen et al., 2016): bilateral primary and secondary somatosensory cortices, bilateral ACC, bilateral insula, bilateral thalamus, and brainstem (Sun et al., 2023, Lançon and Séguéla, 2023, Brooks and Tracey, 2007, Jensen et al., 2016, Groh et al., 2018, Stroman et al., 2018). This approach enabled sampling of a distributed nociceptive-salience network rather than restricting analyses to specific task-evoked activation clusters.

Freesurfer (Fischl, 2012) was used to create parcellation images of each participant’s segmented pre-processed T1-weighted scans. The extracted ROIs were registered to standard space and then combined into a mask for each participant. These masks were then averaged using voxel-wise mean to create a pain pathway mask for ROI focused Fixel-based analysis.

Ten million streamlines passing through the ROI network mask were generated using iFOD2 for each participant. SIFT was performed with a factor of 10, and the resulting million streamlines were used in structural connectivity (SC) matrix generation for each participant. Additionally, Fixel group comparison analysis was performed by using iFOD2 and SIFT on the population template with the ROI network mask. Analysis of FD, LogFC, and FDC followed the same steps as whole-brain analysis.

### 2.7 Graph theory and NBS

The resulting node assignments from SC matrix generation were processed to calculate centroid nodes. Graph metrics were computed across a continuous range of proportional density thresholds from 10% to 30% in 5% increments. For each node and edge, the Area Under the Curve (AUC) was calculated to provide a single, robust scalar metric integrated across densities.

These centroid nodes and edges were then used to calculate graph theory metrics with Matlab Brain Connectivity Toolbox (Rubinov and Sporns, 2010). Following a published systematic review of graph theory in pain neuroimaging (Lenoir et al., 2021); integration was measured by global efficiency, which reflects an inverse of the shortest path to all areas in the network. Segregation was measured by local efficiency, which reflects how well the area is connected to its neighbours. Centrality was measured by node degree, which compared the number of connections to the area, and betweenness centrality, which compared how many shortest paths connect through the area.

Efficient local structural connectivity in human brain networks tend to combine high integration (global efficiency) with high segregation (local efficiency). The connections between nodes are referred to as ‘edges’, and their analysis can provide complementary information on centrality and strength of neural connection coming from nodes. Edges were measured by edge betweenness.

Statistical significance was determined using non-parametric permutation testing of 5,000 iterations. To control for multiple comparisons, the false discovery rate (FDR) correction was applied across all nodes and edges. Effect sizes were calculated with Hedges’ g alongside 95% bootstrapped confidence intervals with 1,000 resamples.

**Fig. 1:**
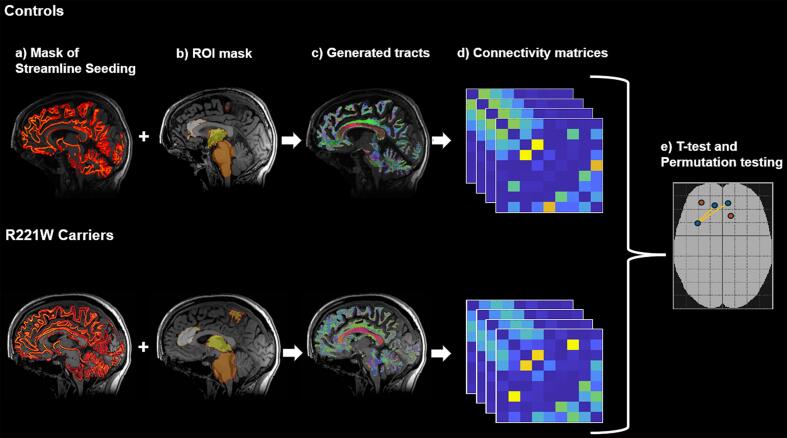
Process of (a) creating anatomically constrained white matter mask with (b) region of interest (ROI) mask to generate (c) Fixel tracts. These tracts then produced (d) connectivity matrices for both groups that were (e) compared using NBS *t*-test and permutation testing.

Edge strength was analysed using Network-Based Statistics (NBS) (Xia et al., 2013). NBS constitutes nonparametric testing that controls for family-wise error rate and uses 5000 permutations with *t*-test thresholds set at 2.5, 3.0, 3.5, and 4.0. The significant P < 0.05 was corrected for multiple comparisons using NBS correction. The NBS process is illustrated in Fig. 1.

## 3 Results

### 3.1 Whole-brain Fixel-based analysis results

**Fig. 2:**
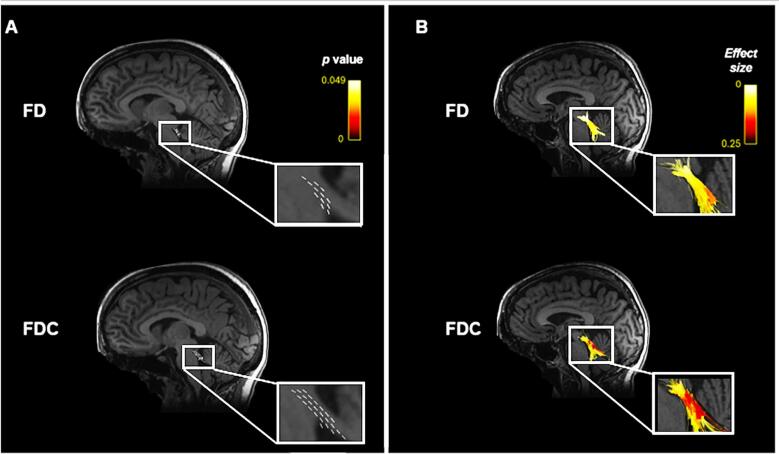
A) Fibre Density (FD) and Fibre Density and Cross-section (FDC) Fixel plots coloured by significance p overlayed over the population white matter template. B) Streamlines coloured by effect side passing through significant Fixel plot FD and FDC areas overlayed over T1 scans.

Fixel-based group comparison between R221W carriers and healthy controls showed significantly reduced FD and FDC in midbrain and pons of R221W carriers (p < 0.05) (Fig. 2), but no significant differences in LogFC. Overlaying the results on a JHU atlas-based white-matter provided specificity in identifying tracts with significantly reduced FD and FDC of R221W carrier group as compared to healthy controls. These affected tracts were: the middle cerebellar peduncle, corticospinal tract and medial lemniscus (both associated with corticospinal pathways), corona radiata, external capsule, and inferior and superior cerebellar peduncles. FDC revealed only differences in tracts connecting the internal capsule, and uncinate fasciculus. FD and FDC Fixel plots with significance *p* and streamlines, which are coloured according to effect sizes, are visualised in Fig. 2.

### 3.2 ROI Fixel-based analysis results

**Fig. 3:**
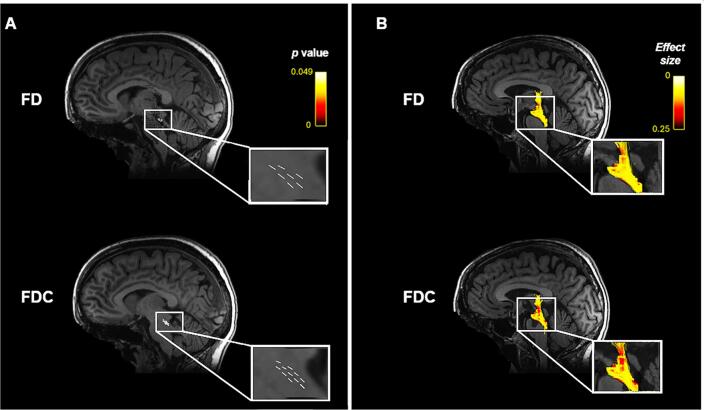
A) Significant Fixel plot Fibre Density (FD), and Fibre Density and Cross-section (FDC) areas overlayed over the population white matter template. B) Streamlines coloured by effect sizes passing through significant Fixel plot FD and FDC areas.

The whole-brain analysis results were replicated in cortical pain-associated ROIs. The Fixel-based group comparison found significantly reduced FD and FDC in the midbrain and pons of R221W carriers (p < 0.05). There were no significant LogFC differences. When streamlines passing through significant FD and FDC plots were overlayed over the JHU white matter atlas, most tracts mirrored whole-brain analysis − the corticospinal tract, medial lemniscus, inferior and superior cerebellar peduncles. Additionally, ROI analysis identified significant FD and FDC reduction in the pontine crossing tract. FD and FDC Fixel plots, as well as streamlines going through them, are visualised in Fig. 3 with *p* significance and effect size.

### 3.3 ROI graph theory analyses

Moving from streamlines to white matter networks, between-group differences in ACC were identified. All reported graph metrics reflect AUC-integrated values across the 10% to 30% proportional density threshold range to provide a single, robust scalar metric. There were no significant differences (p = 0.115) in AUC-integrated global efficiency scores between control (E_glob_ = 0.063) and R221W carriers (E_glob_ = 0.068) in higher pain processing network areas. With respect to segregation of white matter networks, AUC-integrated local efficiency was not significantly different between R221W carriers and controls. R221W carriers showed significantly lower betweenness centrality (reflecting the number of shortest paths through a given node) in left ACC, compared to controls. AUC-integrated node degree (reflecting the number of edges connecting to a given node) was significantly lower in the left ACC of R221W carriers when compared to controls. The significant AUC-integrated graph metric scores are presented in Table 1, and all graph theory metric results are presented in Supplementary Table 1*.*

**Table 1:** Significant group differences across AUC-integrated graph metrics.

**ROI / Connection**	**Metric**	$P_{perm}$**^a^**	$P_{FDR}$**^b^**	**Hedges'**g**^c^**	**95% CI**
Left ACC^d^	Node Degree	< 0.001*	0.004*	−1.96	[-3.35, −1.22]
Left ACC	Betweenness Centrality	< 0.001*	0.004*	−1.90	[-3.34, −1.29]
Right ACC and Right Thalamus	Edge Betweenness	< 0.001*	0.007*	2.39	[1.63, 3.93]
Left ACC and Left Insula	Edge Betweenness	0.001*	0.025*	−0.76	[-1.34, −0.51]
Left ACC and Right Thalamus	Edge Betweenness	0.004*	0.048*	−1.42	[-2.75, −0.79]

a − uncorrected permutation p-value; b – false discovery rate; c – effect size with negative g indicating reduced connectivity in R221W carriers (Patients < Controls and positive g indicates increased connectivity in carriers (Patients > Controls); d – Anterior Cingulate Cortex. * indicates statistical significance p < 0.05.

**Fig. 4:**
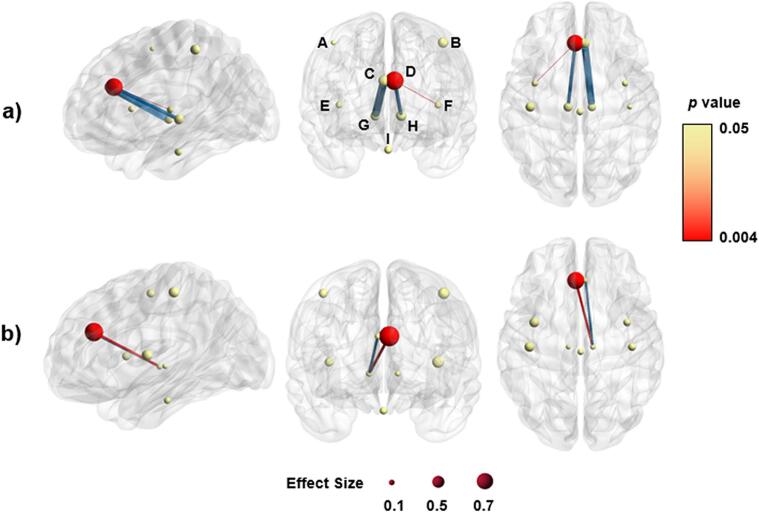
Visualisation of graph theory and NBS comparisons. (a) represents significant betweenness centrality and edge betweenness of R221W carriers and control groups; (b) represents significant node degree and NBS connections of R221W carriers and control groups. Node size represents effect size and the colour represents their p-value, while edges are presented as blue or red based on positive or negative effect respectively. Region labels: (A) sensory cortex right, (B) sensory cortex left, (C) ACC right, (D) ACC left, (E) insula right, (F) insula left, (G) thalamus right, (H) thalamus left, (I) brainstem. This figure was made using BrainNet Viewer (Xia et al., 2013). (For interpretation of the references to colour in this figure legend, the reader is referred to the web version of this article.)

Alongside node differences, AUC-integrated edge metrics provided additional information about connectedness between ROI areas. The mean AUC-integrated edge betweenness score for controls was *b_e_* = 1.486 (SD = 1.761) and for R221W carriers was *b_e_* = 1.360 (SD = 1.408). In R221W carriers, edge betweenness was significantly weaker as compared to controls in left ACC connection to left insula (*p =* 0.025) and right thalamus (*p* = 0.48), but significantly stronger in the connection between right ACC and right thalamus (*p* = 0.007). AUC-integrated edge betweenness, node degree, and betweenness centrality are illustrated in Fig. 4.

Node and edge metrics were shown to be directionally consistent and medium-to-large across the 10% to 30% proportional density range (Supplementary figure 1*)*. While some edge differences were only seen at densities of 20% and above due to network pruning at sparser thresholds, the magnitude of all significant group differences remained stable.

### 3.4 ROI NBS analysis

NBS was performed twice: first to test connectivity of the control group as compared to R221W group, and the opposite comparison for R221W group, all 8 (two sets of 4 t-tests) tests were then corrected for FDR. Similar to graph metrics, both results found significant differences in the thalamus between groups. The R221W group showed stronger connection between the right ACC and right thalamus compared to controls (Table 2). This difference was only significant at *t*-test threshold of 3.0. The R221W group did show significantly worse connectivity from right thalamus to left ACC and to left insula at all *t*-test thresholds (Table 3*)*. The findings are represented in Fig. 4.

**Table 2:** Network Based Statistics comparison of R221W carrier group against control group carrier group at different *t*-test thresholds, multiple comparison corrected.

**Threshold**	2.5	3.0	3.5	4.0
***p* value**	0.124	0.048*	1.000	1.000

Significant differences are indicated by *.

**Table 3:** Network Based Statistics comparison of control group against R221W at different *t*-test thresholds, multiple comparison corrected.

**Threshold**	2.5	3.0	3.5	4.0
***p* value**	0.019*	0.003*	0.016*	0.004*

Significant differences are indicated by *.

## 4 Discussion

This study provides first evidence that the R221W mutation of the Nerve Growth Factor (NGF) gene is associated with differences to the integrity of nerves in the central nervous system, and not just the documented peripheral nervous system alterations. Whole brain and ROI Fixel analyses identified fibre density and cross-section reduction in a number of sensorimotor, but primarily motor, white matter tracts passing through the pons and midbrain of R221W carries. The differences being constrained to the brainstem is an important finding as the brainstem is an integral area for processing nociceptive stimulus and affective-motivational pain response (Petrovic et al., 2004, Stroman et al., 2018). These efferent-related alterations in brainstem white matter run contrary to our original hypothesis that R221W carriers would display afferent-related spinocortical tract deficits, but adds to accumulating evidence that motor (Perini et al., 2020), rather than simply somatosensory, differences are integral to the R221W phenotype.

Although no group differences were found in global network efficiency, which quantifies information integration over the larger white matter network, local network efficiency differences were found in nociceptive and salience subnetworks including ACC and insula. These differences in R221W carriers suggest that inefficient connectivity among these nodes may bias processing to parts of the network more related to somatomotor than affective-motivational aspects of behavioural responses to acute pain.

### 4.1 NGF and the R221W neural phenotype

NGF is primarily constrained to the peripheral nervous system and research on NGF impact on the central nervous system has been limited (Barker et al., 2020). Rat cell line models suggest that the missense point mutation involved in the R221W phenotype affects cleavage of pro-NGF in intracellular pathways, limiting the availability of functional mature NGF in the extracellular space (Carvalho et al., 2011, Larsson et al., 2009). The R221W carriers’ reduced unmyelinated C afferent density may result from insufficient trophic support during development, with possible additional effects of altered regulatory signaling in NGF-mediated nociceptive pathways in adulthood. The trophic role of NGF likely does not substantially affect large myelinated Aβ fibre axons, the cell bodies of which are negative for the primary NGF receptor TrkA (Patapoutian and Reichardt, 2001). However, For brainstem fibre tracts, NGF differences could be coupled with transneural degeneration as a consequence of fewer C- and A-delta fibre inputs (Berry et al., 2012, Shelton and Reichardt, 1986, Basbaum et al., 2009) − second-order neurons that synapse with C-nociceptor fibres in spinal lamina I and extend to parts of the brainstem (Corbetta et al., 2005). Since C-nociceptor fibres are reduced in R221W carriers (Perini et al., 2020), congenital disruption of second-order afferent synapses is propagated to downstream connectivity alterations in brain networks (Fornito et al., 2015, Mufson et al., 1999). These biological changes could influence motor fibres in the brainstem and cortex, and possibly contribute to the reduced behavioural response to pain of R221W (Perini et al., 2016).

### 4.2 Somatomotor and affective-motivational network differences in R221W

A majority of the tracts with reduced fibre density and cross section connect the midbrain to the cerebellum and relate to movement coordination: the superior cerebellar peduncle (Hüttlova et al., 2014), middle cerebellar peduncle (Wang et al., 2014), and the pontine crossing tract (Zhang et al., 2015). Additionally, effects on corticospinal tract (Jang, 2009) but not spinothalamic tracts could suggest that the altered behavioural response can be attributed to affected white matter motor efference rather than somatosensory afference. The two other affected tracts, medial lemniscus and inferior cerebellar peduncle, both relate to proprioceptive information relay (Chilvers et al., 2022, Pijnenburg et al., 2014) from the peripheral nervous system to the somatosensory cortex and brainstem, respectively. This information is not nociceptive but could be involved in coordinating motor behaviour in response to pain (Lin et al., 2019). These tract differences could relate to the dampened motivated responses of R221W carriers in reaction to painful stimulation (Perini et al., 2020). This and previous results are consistent with a transgenic mouse model of the human R221W (or R100W) mutation, which displays behavioural under-reaction and increased response latencies to noxious stimuli, alongside decreased neural activation in motor cortex and striatum (Perini et al., 2021, Testa et al., 2019).

The medial lemniscus projects largely via the ventral posterior inferior thalamus nucleus to primary somatosensory cortex (Chilvers et al., 2022). R221W carriers show reduced fibre density and cross-section here, and this disruption appears to extend into the topology of the cortical network. Specifically, the Left ACC of R221W carriers showed significantly fewer connections with wider network nodes and reduced betweenness centrality compared to controls. Unlike the somatosensory cortices, which maintained nodal connectivity levels similar to controls, the Left ACC appears structurally isolated, participating in fewer shortest-path communications across the network. This reduction suggests that the affective-motivational hub of the pain network is less structurally engaged, potentially forcing a reliance on the preserved primary somatosensory and motor cortices during acute pain processing.

R221W carriers’ ACC also exhibits differences in integration with wider cortical network regions, specifically regarding laterality. Edge-level analysis and NBS results revealed that carriers have significantly weaker connectivity between the Left ACC and the Right Thalamus. In contrast, the connection between the Right ACC and the Right Thalamus was significantly stronger in carriers. This pattern suggests a topological shift: a reduction in cross-hemispheric integration co-occurring with a relative strengthening of ipsilateral connectivity. These laterality differences and interhemispheric disruptions may account for the pain response latency seen in the anterior-mid cingulate cortex of R221W carriers (Perini et al., 2020). In previous research, the BOLD response of R221W carriers’ reported urge to move away from a painful thermal stimulus emerged only after a 2–3 s delay following the stimulus onset, which covaried with MCC activation in controls but not carriers (Perini et al., 2020).

### 4.3 Local network reorganisation in the R221W phenotype

The NBS result indicating that R221W carriers have a significantly weaker subnetwork connecting the right thalamus to the left ACC and left insula, in contrast to the strengthened connection between the right thalamus and right ACC, suggesting a compensatory shift not captured in functional MRI research. The structural disconnection of the Left Insula from the thalamus mirrors the isolation of the Left ACC, indicating a broader difference of R221W carriers’ left hemisphere salience network. However, the significantly stronger ipsilateral connectivity between R221W carriers’ Right Thalamus and Right ACC could suggest an increased reliance on the right hemisphere network for pain and salience processing.

This structural laterality aligns with functional observations regarding task differentiation. Stronger hemodynamic activation of right AI was proportional to how well R221W carriers differentiated between task-relevant and task-irrelevant painful and nonpainful stimulation (Perini et al., 2020). While the structural connectivity of the left insula appears to be weaker when compared to controls, the functional engagement of the right insula and its potential structural integration with the strengthened right-sided thalamic pathway could indicate compensatory aspects of pain behaviour.

However, a pain processing and salience network that is more asymmetric and reliant on an ipsilateral subnetwork may be insufficient for driving the affective-motivational aspects of pain typically associated with the left hemisphere (Perini et al., 2020). This potential compensation, operating alongside the reduced motor cortex engagement, may contribute to inefficient network processing and the attendant latencies in behavioural responses (Pijnenburg et al., 2014). It is also possible that the reduced integration of the left insula in this subnetwork renders the salience system less efficient for producing quick and adaptive behaviour. While cross-hemispheric thalamus-insula connectivity cannot fully compensate for impaired Left ACC integration, the right-sided subnetwork could support downstream signalling among cortical areas responsible for the selection and preparation of responses to noxious stimuli (Labrakakis, 2023, Fuchs et al., 2014, Yen and Lu, 2013), albeit in a potentially less efficient or rapid manner.

### 4.4 Limitations and future directions

One of the inherent limitations of a rare R221W mutation is a small sample size, which limits statistical investigations. address the potential bias of arbitrary threshold selection in a small sample, we calculated the Area Under the Curve (AUC) for graph metrics across a continuous range of proportional density thresholds (10–30%) rather than relying on a single density. This approach was necessary to ensure comparability across participants by controlling for individual differences in edge density and providing a robust, integrated scalar metric (Jenkinson and Beckmann, 2012, Tournier et al., 2012). However, it is acknowledged that this method may still overlook weaker but potentially significant connections outside this range, which could be explored in future studies using alternative approaches, such as fully weighted or density-preserving methods.

The diffusion acquisition of this study was single shell low-angular resolution data, which is below the angular resolution and multi-shell acquisitions generally recommended for optimal FBA. Furthermore, the imaging parameters utilized relatively unconventional anisotropic voxel dimensions (0.5 × 0.5 × 1 mm for T1-weighted and 1 × 1 × 2 mm for DWI). While the higher-resolution T1-weighted images beneficially mitigate partial volume effects and reduce uncertainty during ROI definition and parcellation, the anisotropic nature of the diffusion data remains an inherent limitation that could influence streamline tractography and connectome generation. However, the SS3T-CSD used in our pipeline is able to mitigate this by ensuring robust estimation of white matter FODs. SS3T-CSD has been shown to produce stable and biologically meaningful fibre orientation estimates in datasets that do not meet optimal multi-shell acquisition standards, and has demonstrated good test–retest reliability (Dhollander et al., 2021, Newman et al., 2019). Additionally, while our graph theory analysis was constrained to a hypothesis-driven network of nociceptive and salience regions, this targeted approach allowed for specific investigation of relevant pathophysiology. Future studies with larger multi-shell acquisitions, isotropic acquisitions and whole-brain parcellations would strengthen confidence in these edge-level findings and potentially uncover wider network effects.

While the R221W gene mutation group was small, the breadth and impact of these results could have implications for other peripheral neuropathy and pain indifference populations. Convergent findings across FBA, graph metrics, and NBS strengthen confidence in the results but further research on other CIP groups, such with mutations in SCN9a (Cox et al., 2006), CLTCL1 (Nahorski and Al-Gazali, 2015) or NTRKI (Indo, 2001), is needed to compare pain insensitivity mechanisms between different congenital conditions, and to compare groups with CIP to acquired peripheral neuropathy on structural brain white matter differences. This comparison is particularly needed as white matter reorganisation has been seen in other congenital sensory loss groups, but it has been found to be significantly different to groups who acquired sensory loss later in life (Wang et al., 2013, Simon et al., 2020).

Future research should further examine the potentially disrupted cross-hemispheric connection, and its relation to the affective-motivational networks. The comparison of peripheral neuropathy with CIP due to central nervous system changes would also provide more insight of different CIP mechanisms.

### 4.5 Conclusions

This study presents three different white matter analysis frameworks that resulted in nuanced findings of structural disruption and altered topology in people with CIP. Whole-brain and ROI Fixel-based analyses identified a number of altered R221W carriers’ sensorimotor pathways that could contribute to the reduced behavioural response to pain of R221W carriers. Crucially, as the sensory motor and spinothalamic network integrity remained intact, these findings support a previously proposed theory (Perini et al., 2020) that CIP in R221W carriers is one of motor under-reactivity and pain indifference, rather than pain insensitivity.

Graph theory and NBS results further revealed that while the wider network maintains global efficiency, the motivation-related Left ACC appears structurally isolated, showing significantly reduced nodal degree and centrality. Rather than a generalized reduction in quality, the analysis identified distinct structural differences suggesting potential local and cross-hemispheric compensatory reorganisation. Specifically, the topological shift toward a stronger right-hemisphere thalamic connection suggests a mechanism for preserving pain discrimination despite the degradation of the left-hemisphere affective-motivational loop.

Currently, these findings do support the integrative role of the thalamus, ACC, and insula in somatosensory and motor reactivity to nociception (Labrakakis, 2023, Fuchs et al., 2014, Yen and Lu, 2013) by showcasing how their disruption can lead to signs of impaired pain processing and reactivity. While there is no previous research of peripheral CIP somatosensory symptoms translating to central nervous system changes, chronic pain neuropathy conditions provide comparable findings of reduced structural integrity in the somatosensory cortex (Selvarajah et al., 2019, Teh et al., 2021) and ACC (Zhang et al., 2023). These structural findings require further clinical and neuroimaging testing in both CIP and painful peripheral neuropathy populations to understand the full impact of peripheral neuropathy on central nervous system reorganisation. The combined results suggest a complex interplay of disruptions and compensations within pain-processing and motor effector networks, providing support for the theory that congenitally reduced nociceptive fibres can impact the central nervous system development in CIP populations.

## CRediT authorship contribution statement

**Arnas Tamasauskas:** Writing – review & editing, Writing – original draft, Visualization, Project administration, Methodology, Formal analysis, Conceptualization. **Irene Perini:** Writing – review & editing, Resources, Investigation, Funding acquisition, Data curation, Conceptualization. **Jan Minde:** Investigation, Conceptualization. **Simon S. Keller:** Writing – review & editing, Supervision. **Nicholas Fallon:** Writing – review & editing, Supervision. **Bernhard Frank:** Writing – review & editing, Supervision. **India Morrison:** Writing – review & editing, Validation, Supervision, Project administration, Investigation, Conceptualization. **Andrew Marshall:** Writing – review & editing, Validation, Supervision, Project administration, Funding acquisition, Data curation, Conceptualization.

## Declaration of competing interest

The authors declare the following financial interests/personal relationships which may be considered as potential competing interests: Arnas Tamasauskas reports financial support was provided by UK Research and Innovation Medical Research Council. If there are other authors, they declare that they have no known competing financial interests or personal relationships that could have appeared to influence the work reported in this paper.

## Data Availability

Data is made freely available with CC0 licence on Figshare (Tamasauskas, 2025) and the code is freely accessible on GitHub (Tamasauskas, 2025).
