# Supplementary figures and images for: White matter differences in motor and affective-motivational networks of pain-indifferent carriers of the R221W mutation
Source: Neurobiol Pain. 2026 Mar 28;20:100211. doi: 10.1016/j.ynpai.2026.100211 (PMC13084749; doi:10.1016/j.ynpai.2026.100211)

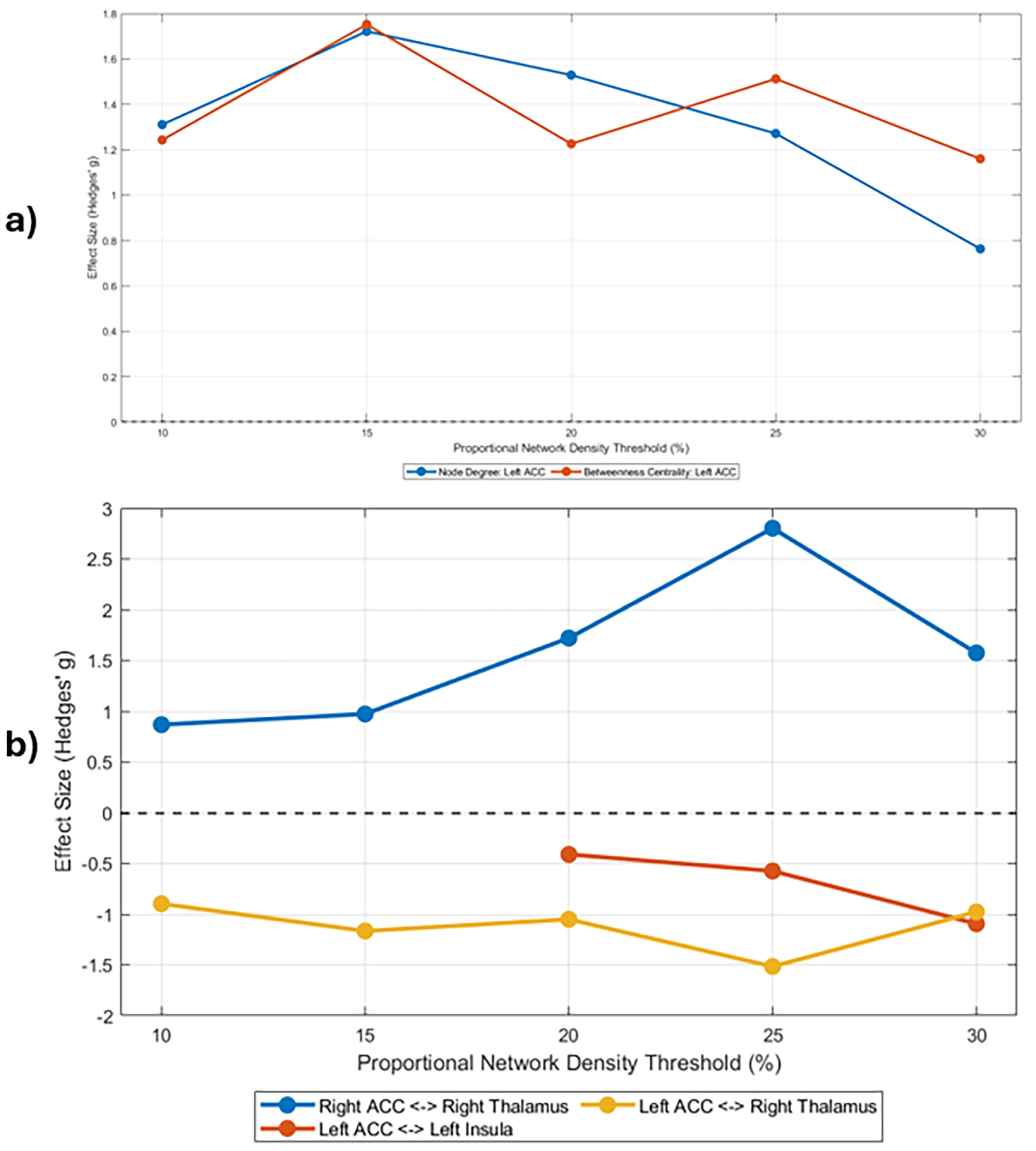

Supplement: Supplementary Figure 1 [file mmc1.jpg]
