# Supplementary material for: White matter differences in motor and affective-motivational networks of pain-indifferent carriers of the R221W mutation
Source: Neurobiol Pain. 2026 Mar 28;20:100211. doi: 10.1016/j.ynpai.2026.100211 (PMC13084749; doi:10.1016/j.ynpai.2026.100211)
Supplement: Supplementary Data 2 [file mmc2.docx]

***Supplementary Table 1: Group differences across graph metrics***

| ***ROI*** | $P_{perm}$ **^a^** | $P_{FDR}$ **^b^** | ***Hedges'***$g$**^c^** | ***95% CI*** |
| --- | --- | --- | --- | --- |
| ***Node Degree*** |  |  |  |  |
| ***Left ACC***^d^ | ***< .001****** | ***.004****** | ***-1.96*** | ***[-3.35, -1.22]*** |
| *Left Insula* | *.063* | *.253* | *-0.80* | *[-1.64, -0.06]* |
| *Left S1^e^* | *.084* | *.253* | *-0.76* | *[-1.84, 0.00]* |
| *Right S1* | *.266* | *.572* | *0.46* | *[-0.38, 1.29]* |
| *Right Insula* | *.318* | *.572* | *0.41* | *[-0.40, 1.32]* |
| *Brainstem* | *.573* | *.859* | *-0.24* | *[-1.06, 0.58]* |
| *Right Thalamus* | *.837* | *.993* | *0.09* | *[-0.82, 0.97]* |
| *Right ACC* | *.882* | *.993* | *-0.06* | *[-0.78, 0.97]* |
| *Left Thalamus* | *.999* | *.999* | *0.00* | *[-0.81, 0.97]* |
| ***Betweenness Centrality*** |  |  |  |  |
| ***Left ACC*** | ***< .001****** | ***.004****** | ***-1.90*** | ***[-3.34, -1.29]*** |
| *Right ACC* | *.042* | *.190* | *0.90* | *[0.12, 2.58]* |
| *Left Thalamus* | *.086* | *.225* | *0.75* | *[0.00, 2.05]* |
| *Left S1* | *.100* | *.225* | *-0.76* | *[-1.54, -0.12]* |
| *Right Thalamus* | *.149* | *.269* | *0.63* | *[-0.11, 1.59]* |
| *Brainstem* | *.364* | *.546* | *-0.42* | *[-1.11, 0.49]* |
| *Left Insula* | *.476* | *.611* | *-0.30* | *[-1.22, 0.57]* |
| *Right Insula* | *.546* | *.614* | *0.25* | *[-0.58, 1.14]* |
| *Right S1* | *.910* | *.910* | *0.05* | *[-0.98, 0.86]* |
| ***Local Efficiency*** |  |  |  |  |
| *Right Insula* | *.270* | *.867* | *0.46* | *[-0.30, 2.10]* |
| *Left S1* | *.295* | *.867* | *-0.48* | *[-1.31, 0.29]* |
| *Left ACC* | *.323* | *.867* | *-0.54* | *[-1.05, 0.67]* |
| *Left Insula* | *.416* | *.867* | *-0.32* | *[-1.31, 0.46]* |
| *Left Thalamus* | *.482* | *.867* | *-0.31* | *[-1.07, 0.55]* |
| *Right Thalamus* | *.828* | *1.000* | *-0.12* | *[-0.75, 1.34]* |
| *Brainstem* | *.871* | *1.000* | *0.08* | *[-0.83, 0.89]* |
| *Right ACC* | *.987* | *1.000* | *0.01* | *[-0.75, 1.81]* |
| *Right S1* | *1.000* | *1.000* | *-0.41* | *[-0.79, -0.41]* |

*a - uncorrected permutation p-value; b – false discovery rate; c – effect size with negative g indicating reduced connectivity in R221W carriers (Patients < Controls and positive g indicates increased connectivity in carriers (Patients > Controls); d – Anterior Cingulate Cortex; e – sensorimotor cortex. * indicates statistical significance p < 0.05.*
